# Mechanical Nitriding of Titanium and Its Alloys as a Feedstock for the Additive Manufacturing of Functionally Graded Materials

**DOI:** 10.3390/ma19061115

**Published:** 2026-03-13

**Authors:** Anna Antolak-Dudka, Malwina Liszewska, Sławomir Dyjak, Iwona Wyrębska, Tomasz Czujko, Marek Polański

**Affiliations:** 1 Institute of Materials Science and Engineering, Military University of Technology, Kaliskiego 2, 00-908 Warsaw, Poland; anna.dudka@wat.edu.pl (A.A.-D.); iwona.wyrebska@wat.edu.pl (I.W.); marek.polanski@wat.edu.pl (M.P.); 2 Institute of Optoelectronics, Military University of Technology, Kaliskiego 2, 00-908 Warsaw, Poland; malwina.liszewska@wat.edu.pl; 3 Institute of Chemistry, Military University of Technology, Kaliskiego 2, 00-908 Warsaw, Poland; slawomir.dyjak@wat.edu.pl

**Keywords:** titanium nitride, reactive milling, self-shearing, functionally graded biomaterials

## Abstract

**Highlights:**

- TiN coatings were obtained on the surface of the titanium and its alloys powders.
- Self-shearing reactive milling under nitrogen pressure was performed.
- The thickness of TiN coatings was in a nanometric scale.
- No grinding media was used during the milling processes.
- The spherical shape of the powder particles after milling was preserved.

**Abstract:**

This work focuses on obtaining a titanium nitride coating on the surfaces of titanium and its alloy powders using a novel method, self-shearing reactive milling, under a nitrogen pressure of 50 bar. The Ti, Ti6Al4V, and Ti-5553 spherical powders were milled for up to 10 h at ambient temperature without grinding balls. As a result of the experiments, a thin, brittle TiN coating formed on the powders’ surfaces. The cross-sections of the milled powders reveal that the TiN layer thickness is in the nanometer range (about 500 nm). By analyzing the sequence of X-ray diffraction patterns, it is evident that only for the Ti6Al4V powder milled for 10 h, two peaks are observed that can be attributed to a TiN phase. On the other hand, Raman spectroscopy revealed characteristic TiN spectra even for samples collected at the initial stage of self-shearing reactive milling. An important aspect of the experiment was the preservation of the spherical shape of the milled powders, which makes them a potential feedstock for additive manufacturing of functionally graded biomaterials.

## 1. Introduction

Titanium and its alloys are widely used across various industries, including medicine and aerospace, due to their exceptional strength-to-density ratio, corrosion resistance, and biocompatibility [1]. The classification of pure titanium and titanium alloys is primarily based on their microstructural characteristics, which define three main groups: single-phase α alloys or β alloys, and dual-phase α + β alloys [2]. Each category exhibits distinct physical and mechanical properties, making them suitable for specific applications across various industrial sectors [3,4,5,6]. In recent years, titanium alloys have attracted significant interest as feedstock materials for additive manufacturing technologies [7,8,9]. For titanium and its alloys to be effectively utilized in metal additive manufacturing processes, particularly in powder bed fusion (PBF) and directed energy deposition (DED) techniques, they must be provided in the form of powders with a spherical particle morphology to ensure optimal flowability and packing density of manufactured products [10,11,12]. As with all manufacturing methods, additive manufacturing technologies also present specific challenges and limitations, particularly concerning the durability of titanium components and alloys. The surfaces of additively manufactured parts often exhibit insufficient hardness, increased susceptibility to oxidation, and accelerated wear, which can limit their performance in demanding applications [13,14]. Therefore, to improve surface properties such as hardness, wear resistance, and corrosion resistance, various surface coatings are applied, which are specifically engineered to enhance these characteristics and extend the functional performance of titanium components produced by additive manufacturing [15,16,17].

Titanium nitride (TiN) is among the most widely applied coating materials, valued for its high hardness and tribological performance, particularly its tendency to reduce friction when sliding against typical engineering materials. TiN also exhibits excellent wear resistance and a high melting point of approximately 2950 °C, which, combined with its chemical and thermal stability, makes it particularly effective in enhancing the surface of components, especially in applications requiring improved durability (cutting tools), corrosion resistance, and extended service life (biomedical applications) [18,19].

Titanium nitride coatings can be produced on the surface of titanium and its alloys using several techniques such as chemical vapor deposition (CVD) [20], physical vapor deposition (PVD) [21], plasma nitriding [22,23,24,25], ion-beam implantation [26], laser gas nitriding [19,22,27,28,29] or magnetron sputtering [30,31]. Using these techniques, it is possible to obtain an adherent TiN layer with thicknesses ranging from several tens of nanometers to several tens of micrometers, depending on the type of deposition process. The coating’s roughness and adhesion to the substrate may also vary. However, these methods have certain limitations, including high processing temperatures, aggressive chemical environments, and increased operational costs due to the need for advanced equipment, such as systems capable of maintaining high-vacuum conditions. Additionally, conventional TiN deposition methods are generally limited to solid substrates. This has created a demand for alternative techniques capable of synthesizing titanium nitride under milder conditions, particularly methods applicable to powder materials.

An alternative technique could be mechanochemical ball milling, in which the energy generated during powder milling is used to drive chemical reactions and form new materials in solid–liquid or solid–gas systems [32]. The milling process is carried out using grinding balls, which induce a continuous cycle of cold welding and fracturing of powder particles due to the accumulation of a critical number of defects and dislocations within the material, leading to the formation of metastable phases, or even amorphous and nanocrystalline materials. This also leads to a change in the morphology of the powder particles from their initial shape, often resulting in refinement and the formation of flake-like particles. Over the years, several studies have examined the formation of titanium nitrides via mechanochemical ball milling with different nitrogen-donating materials and experimental procedures. Zhang et al. [33] mechanochemically alloyed elemental titanium powder with pyrazine (a nitrogen-containing organic compound) in benzene solution for up to 336 h in a horizontal Uni-Ball Mill with stainless steel balls. During wet milling, the phase Ti_2_N was formed, transforming into TiN upon heating. Another route was to investigate the formation of TiN via mechanical alloying using high-energy ball milling with nitrogen atmosphere [34,35,36]. Calka et al. [35] found that the TiN formed after 36 h of mechanochemical synthesis, and a fully converted TiN phase was detected by X-ray diffraction after 60 h of milling. There have also been attempts to obtain titanium nitride via mechanochemical alloying with grinding balls, using ammonia (in liquid or gaseous form) as the nitrogen source [37,38]. In the following stages, TiN powders can be consolidated [39] and even used to manufacture Ti-TiN composite materials, as Wexler described in his previous works [37,40].

All the experiments mentioned above used steel balls as the grinding medium, which changed the shape of the powder particles. Unfortunately, one of the important requirements for powders used in additive manufacturing, apart from appropriate granulation, is their sphericity. This prompted us to look for an alternative method that would allow the powder to retain its original spherical shape. It was found that the formation of a new chemical compound can be induced by mechanochemical synthesis at room temperature, without grinding balls. Patel et al. [41] investigated two methods for hydrogenating FeTi powder: conventional reactive ball milling (RM) under hydrogen pressure using steel grinding balls in a planetary mill and another novel method called self-shearing reactive milling (SSRM), where the powder was milled without grinding media, relying only on particle–particle and particle–wall collisions. It was surprising that applying SSRM resulted in complete hydrogenation of FeTi powder at room temperature, without grinding balls. A similar phenomenon was observed by Pistidda et al. [42], who reported the synthesis of Mg(BH_4_)_2_ during the milling of MgB_2_ powder under hydrogen pressure, without grinding media. Hydrogenation of titanium and its alloys (α, α + β, β) powders using the self-shearing reactive milling was investigated and reported by Wyrębska et al. [43]. In all cases, during spontaneous hydrogenation, stoichiometric TiH_2_ has formed, and the reaction has continued even without further milling. In light of those results, it became clear that the normally underestimated kinetic energy of powder particles colliding with vial walls and with each other is sufficient to initiate chemical reactions, especially with gas substrates under pressure.

Encouraged by previous literature, the authors present a novel approach to produce titanium nitride (TiN) during self-shearing reactive milling without grinding balls, using spherical Ti, Ti6Al4V, and Ti-5553 alloy powders under nitrogen pressure at ambient temperature. It was found that, using this method, TiN can be formed on the surface of powder particles by a controlled reaction between nitrogen and titanium. Preservation of particle morphology is an additional point of interest, opening the possibility of using these surface-modified powders in additive manufacturing of functionally graded biomaterials.

## 2. Materials and Methods

Reactive self-shearing milling processes were conducted under nitrogen pressure for titanium and titanium alloys powders using a Pulverisette P6 (Fritsh GmbH, Idar-Oberstein, Germany) planetary mill without grinding media. The experiments were performed in a custom-made stainless steel vial of 65 mm in height and 70 mm in internal diameter. These vial dimensions enable self-shearing reactive milling of 200 g of metallic powder in a single process cycle, with an effective powder filling degree of ~20%. This is important from the perspective of subsequent use of the reactive-milled powder, because the amount is sufficient for use as an initial material in additive techniques. The temperature and pressure signals within the cylinder were collected every second and monitored remotely using a sensor (model TDWLB-DL, 200 bar range, 0.25% of full-scale accuracy, Transducers Direct^®^, Cincinnati, OH, USA) integrated into the cylinder, enabling real-time Bluetooth wireless transmission. During the experiments, high-purity nitrogen gas (Air Products, 99.9999%, <10 ppb O_2_, <20 ppb H_2_O, and <200 ppb H_2_) was used. The reactive self-shearing milling processes were carried out for three different powders with spherical particles: commercially pure Grade 1 Ti (45–106 μm, Carpenter Additive^®^, Philadelphia, PA, USA), Ti6Al4V (45–150 μm, TLS Technik GmbH & Co., Bitterfeld-Wolfen, Germany), and Ti5Al5V5Mo3Cr marked as Ti-5553 (45–106 μm, AP&C GE Additive Company, Boisbriand, QC, USA) titanium alloys. After filling the cylinder with powder and nitrogen to 50 bar, the reactive milling process was initiated approximately 15 h later to ensure temperature stabilization within the cylinder and verify the entire system’s tightness. The powders were reactively milled/mixed in a nitrogen atmosphere for a total of 10 h at 500 rpm. No grinding balls were used at any stage of the process. Powder samples for further investigation were collected at specific intervals: 5, 15, 30, 60 min, and 10 h. Before nitrogen charging, the cylinder was purged three times with argon and subsequently evacuated to 10^−1^ mbar to minimize residual atmospheric contaminants. Sampling for further material characterization was performed in a glovebox under an inert argon atmosphere to prevent material oxidation.

The flowability of the as-received and self-shearing reactive milled Ti, Ti6Al4V, and Ti-5553 powders was evaluated using a Hall flowmeter. All powders were spherical and free-flowing, allowing the Hall flow test to be performed for each material. The measurements were carried out in accordance with ISO 4490:2018 [44]. The Hall flow rate was determined as the time required for 50 g of powder to flow through the calibrated funnel orifice.

The particle size distribution of the as-received and self-shearing reactive milled Ti, Ti6Al4V, and Ti-5553 powders was determined using an IPS UA particle size analyzer (measurement range: 0.5–200 µm, Kamika Instruments, Warsaw, Poland). The results are presented as volume-based particle size distributions together with the mean particle size (D_v) and the median particle size (D_v50).

Microscopic observations of powder particles and their cross-sections were performed using a dual-beam field emission gun scanning electron microscope FEI Quanta 3D equipped with energy dispersive spectroscopy EDS which allows for chemical composition analysis.

Metallographic powders’ cross-sections were prepared by embedding the powder particles in a thermosetting resin. After that, the mounted samples were ground using 600 and 1200-grit SiC abrasive papers. Next, they were polished with a polyurethane cloth using a colloidal silica suspension and 30% hydrogen peroxide. Some cross-sections were subsequently etched using Kroll’s reagent, while others were analyzed in the as-polished condition.

X-ray diffraction (XRD) analysis was performed using a Rigaku Ultima IV diffractometer (Rigaku, Tokyo, Japan) equipped with Co Kα radiation to determine the phase composition of powders subjected to reactive milling.

Raman spectroscopy was performed to analyze potential changes in the surface composition of powders milled reactively for varying times. The Raman spectra were acquired using a Renishaw in Via Reflex Raman microscope (Renishaw Plc, Wotton-under-Edge, UK) equipped with an EMCCD detector (Andor Technology Ltd., Belfast, UK). The measurements were carried out using laser radiation at 785 nm (power ca. 6 mW on the sample) and a 100× objective lens (N.A. = 0.85, Leica, Leitz-Park in Wetzlar, Germany). The measurement parameters were as follows: acquisition time of 5 s and 20 accumulations at each point. The spectra were acquired from 10 random points. The spectrometer was calibrated using an internal silicon wafer, and the spectrum was centered at 520.5 cm^−1^. All collected spectra were processed using WiRE 5.5.

An elemental analysis (i.e., quantification of nitrogen wt.% content) of the synthesized materials was performed using a Vario EL Cube apparatus (Elementar GmbH, Langenselbold, Germany).

## 3. Results and Discussion

The main goal of this work was to confirm the feasibility of using self-shearing reactive milling to produce nanometric titanium nitride coatings on spherical titanium powder TiGd1 and its alloys Ti6Al4V and Ti5553. This will enable the additive manufacturing of components with gradient-variable nitrogen content.

### 3.1. Reaction of Titanium Powders with Nitrogen

**Figure 1:**
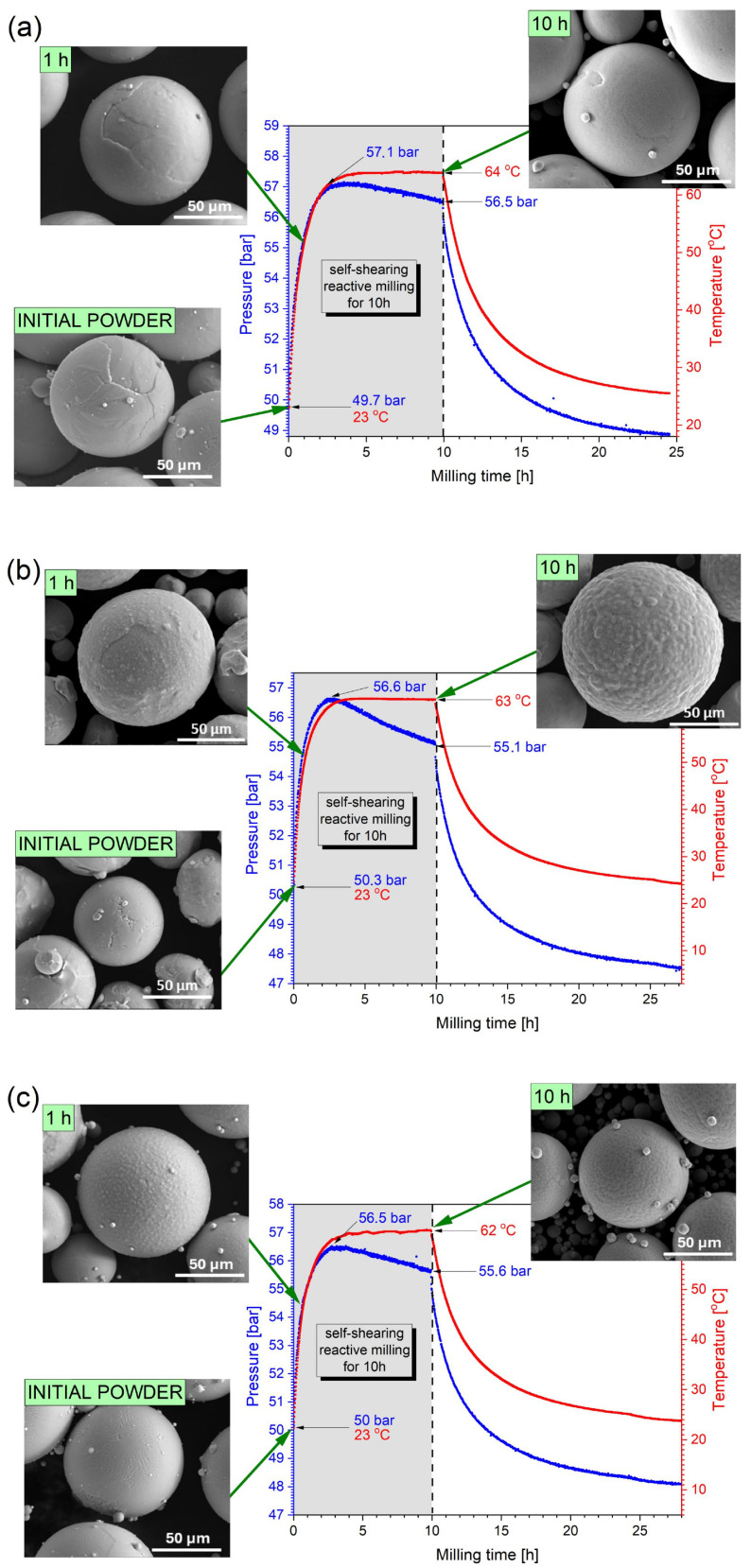
The morphology of initial and milled powders and the diagrams of temperature and nitrogen pressure inside the vial during self-shearing reactive milling processes of (**a**) pure titanium (TiGd1), (**b**) Ti6Al4V, and (**c**) Ti-5553 alloys.

Direct pressure measurement during milling was used as a first method to determine whether nitrogen reacted with titanium. Pressure drop in the vial was expected along with the reaction and possible nitride formation. The changes in temperature and nitrogen pressure inside the vial during self-shearing reactive milling of pure titanium (TiGd1) and its alloys (Ti6Al4V and Ti-5553) without grinding balls are shown in Figure 1. Additionally, Figure 1 shows micrographs of the milled powders to observe how their particle morphology changes over time. In all cases, in the initial stage of self-shearing reactive milling, the temperature inside the vial increases, and the nitrogen pressure follows that. This is most likely attributed to frequent collisions and friction between powder particles and between the powder and the cylinder walls. As a result, the system’s kinetic energy increases, and a significant portion of this energy is converted into heat during collisions, raising the temperature and, consequently, the nitrogen pressure in the vial [41,43]. A significant change in temperature would be expected during an intensive reaction between titanium and nitrogen, given its highly exothermic nature. The standard enthalpy of formation of titanium nitride (TiN) is highly exothermic, generally reported at −324 to −338 kJ/mol at standard conditions [45,46]. After approximately two hours of milling, both the temperature and nitrogen pressure reach their maximum values. The pressure rise indicates a lack of an intensive reaction with nitrogen at this stage. From that point onward, the temperature stabilized at about 62 °C for Ti-5553 and 64 °C for TiGd1 powders, remaining constant until the end of milling, while the nitrogen pressure began to decrease. This pressure drop suggests that nitriding may occur in the milled powders. The largest nitrogen pressure drop during milling, calculated as the pressure decrease from its maximum value to the level measured after 10 h of milling, was observed for the Ti6Al4V alloy powder and amounted to 1.5 bar. It should be noted that calculating the percentage of nitrogen absorbed by milled material using the volumetric method with such a small pressure drop is rather complicated and prone to error. Nevertheless, evaluated according to the Redlich-Kwong equation [43], the adsorbed amount of nitrogen is around 0.3 wt.% for Ti6Al4V powder milled for 10 h. For the remaining powders (TiGd1 and Ti-5553), the nitrogen pressure drop was much lower, at 0.6 and 0.9 bar, respectively. The small pressure drop suggests that, in all cases, nitriding does not occur throughout the entire particle volume but only to a limited extent, most likely on the surface. Although the overall nitrogen pressure drop may not appear particularly large, a comparison of the three titanium and its alloy powders clearly shows that the pressure drop for Ti6Al4V is significantly greater than that for the other two. Consequently, it is expected that the effects of the nitriding process will be more pronounced for this composition.

Compared to the morphology of the initial powders, SEM micrographs of the powder particles (Figure 1a–c) in all cases confirmed that the spherical shape of the particles was preserved throughout the entire self-shearing reactive milling process. Quantitative granulometric analysis of the powders as received and after 10 h of grinding showed that both the particle diameter and its distribution were comparable (Figure S1). Interestingly, the powder flowability as represented in Table S1 improved slightly (TiGd1 and Ti6Al4V) or remained unchanged (Ti5553).

However, despite the overall spherical shape being retained after 10 h of milling, a subtle surface change on the particles could be observed. This effect is particularly visible in the case of the Ti6Al4V powder milled for 10 h, where the particle surfaces appear to exhibit a sponge-like structure with small protrusions. This phenomenon is barely noticeable in the Ti-5553 and TiGd1 powders.

In contrast to the production of nitrided layers by varying the nitrogen concentration in the shielding gas [47], the proposed method for producing powders with a nanometrically thick nitrided layer allows precise control of the nitrogen content in the surface layer of the manufactured element. This will enable the additive manufacturing of functional gradient materials for biomedical applications, with surface modification with titanium nitride.

### 3.2. Chemical and Phase Composition of Milled Powders

**Figure 2:**
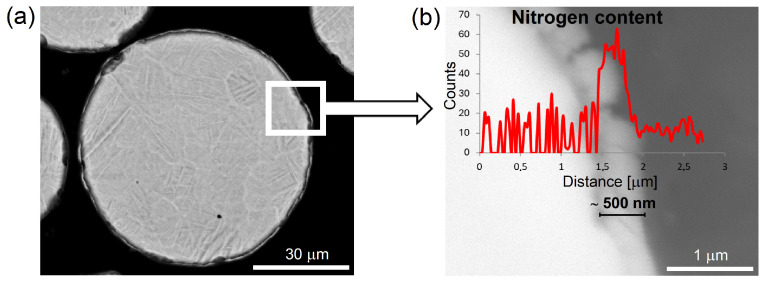
(**a**,**b**) SEM cross-section of Ti6Al4V powder particle after 10 h of self-shearing reactive milling under nitrogen pressure, with (**b**) line scan content of nitrogen analysis.

In order to investigate what phenomena have occurred on the surface of the powder particles and how deep the changes extend, the metallographic cross-sections of the TiGd1, Ti6Al4V, and Ti-5553 powders after 10 h of self-shearing reactive milling were prepared, and an EDS line scan of chemical composition analysis was carried out precisely on these cross-sections. An example of the thickest layer obtained for Ti6Al4V powder milled for 10 h is presented in Figure 2. SEM observations revealed that examining changes on the surface of powder particles is very challenging because the surface layer is extremely thin and brittle, making the preparation of metallographic cross-sections difficult. Despite this, measurements performed on a dozen powder particles (22 measurements) allowed us to estimate the thickness of the nitrided layer. The formed continuous layer has a nanometric thickness (750 ± 270 nm), as seen in Figure 2b, and can be identified as a nitride due to a visible increase in nitrogen content in this layer during the line-scan chemical composition analysis. Assuming stoichiometric composition of titanium nitride TiN and taking into account the average powder particle size 81.4 μm and the average thickness of the nitrided layer, the estimated nitrogen content is about 0.7 wt.%. Given the well-known limitations of the SEM-EDS technique for quantitative analysis of light elements such as nitrogen and the fact that the interaction volume at typical accelerating voltages can exceed 500 nm, we can assume that the measured nitride layer thickness is overstated. For a nitrogen content of 0.3 wt.% determined using the volumetric technique, the average thickness of the obtained layer should be approximately 250 nm.

**Figure 3:**
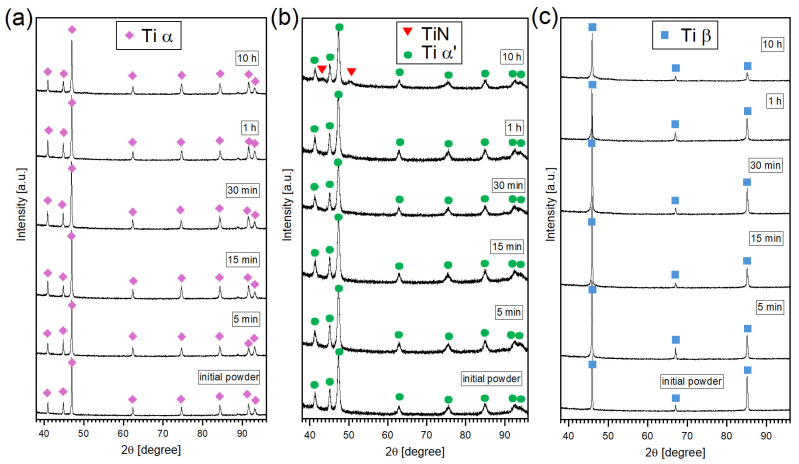
The XRD patterns for different times of self-shearing reactive milling under nitrogen pressure for (**a**) pure titanium (TiGd1), (**b**) Ti6Al4V, and (**c**) Ti-5553 powders.

To verify the formation of titanium nitride on the surface of the powder particles during self-shearing reactive milling, XRD analyses were performed on TiGd1, Ti6Al4V, and Ti-5553 samples subjected to different milling times. The diffraction pattern sequences for all three cases are presented in Figure 3. It was found that in the case of TiGd1 and Ti-5553 powders, no titanium nitride diffraction peaks were identified. Only detectable reflections correspond to the alpha-titanium and beta-titanium phases, respectively, consistent with the fact that TiGd1 is an alpha-titanium alloy while Ti-5553 is a beta-titanium alloy. In the case of Ti6Al4V, the situation is somewhat different. After 10 h of self-shearing reactive milling, the diffractogram reveals not only peaks associated with Ti α’ but also two additional peaks corresponding to TiN (ICDD: 00-038-1420) and to the (111) and (220) crystallographic planes, indicating the formation of titanium nitride [47]. Due to the low amount of TiN formed on the surface of the powder particles, the intensity of the TiN peaks in the case of the Ti6Al4V powder sample is relatively weak, while in the case of the other two compositions, such peaks are not observed at all. Nevertheless, the absence of detectable TiN reflections in the diffractograms does not necessarily indicate the lack of titanium nitride, since the nitride layer may be too thin (as suggested by SEM observations) to produce measurable diffraction peaks.

In most standard powder X-ray diffraction (PXRD) measurements, the safe, practical detection limit for trace phases is considered to be around 1–2 vol.%, below which the minority-phase peaks blend with background noise and become difficult to distinguish. Such a volume fraction of the TiN phase was found only in the case of Ti6Al4V powder after 10 h of grinding

**Figure 4:**
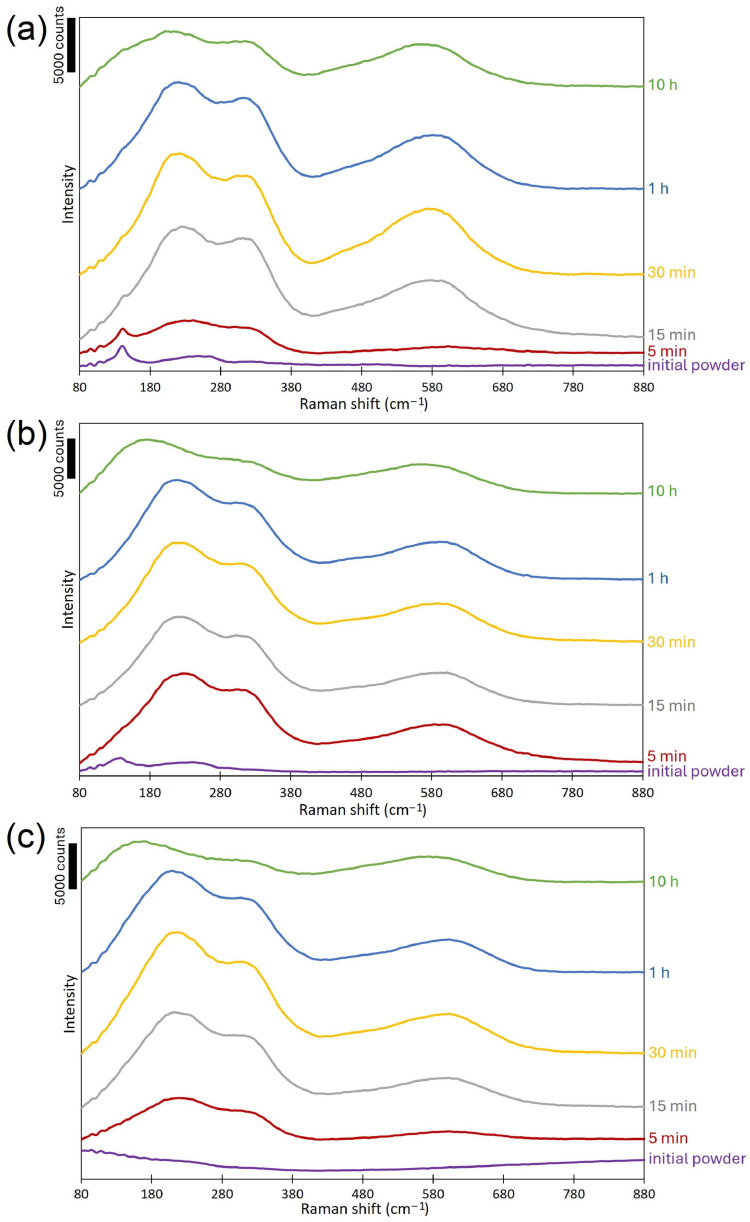
Raman spectra for (**a**) pure titanium (TiGd1), (**b**) Ti6Al4V, and (**c**) Ti-5553 powders subjected to self-shearing reactive milling under nitrogen pressure.

To definitively confirm the formation of a thin TiN layer on the surface of the powder particles during self-shearing milling under nitrogen pressure, Raman spectroscopy was conducted, providing phase-specific identification and characterization of the synthesized material with higher surface sensitivity than deep-penetrating XRD. Figure 4a–c shows Raman spectra of TiGd1, Ti6Al4V, and Ti5553 samples subjected to self-shearing reactive milling under nitrogen pressure for different times, which confirms the presence of titanium nitride. At Table 1, the peak positions are listed for all cases. The Raman lines at 168–246, 299–316, and 564–602 cm^−1^ can be assigned to the transverse acoustic (TA), longitudinal acoustic (LA), and transverse optical (TO) modes of TiN, respectively [48,49,50]. Scattering of acoustic phonons (TA and LA modes) is the effect of Ti ion vibrations. Scattering of optical phonons TO results from nitrogen ion vibrations. The differences in the shift values may result from different arrangements and surroundings of titanium and nitrogen atoms in the TiN crystallographic lattice.

**Figure 5:**
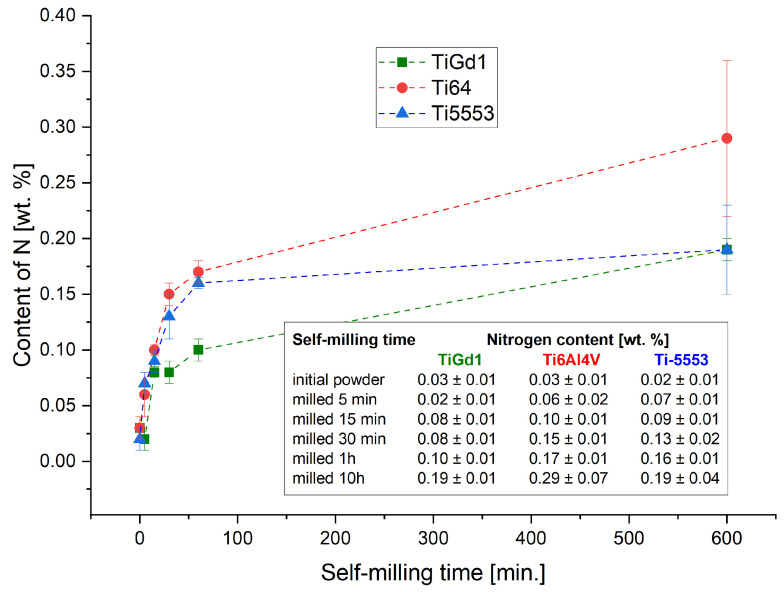
Nitrogen content in Ti, Ti6Al4V, and Ti-5553 powders after self-shearing reactive milling under nitrogen pressure for different milling times.

To determine the nitrogen content in the TiGd1, Ti6Al4V, and Ti-5553 powders after different times of self-shearing reactive milling, elemental analysis was performed, and the results are presented in Figure 5. It is clear that in the early stages of milling (up to 1 h), the nitrogen content in TiGd1 powder is significantly lower than in Ti6Al4V and Ti-5553 powders. Whereas, after 10 h of milling, a pronounced increase in nitrogen content is observed in the Ti6Al4V powder, indicating a more advanced progression of the nitriding process in this material.

According to the results, nitriding of the powder surface during self-shearing reactive milling under nitrogen pressure proceeds more intensively and more distinctly in the Ti6Al4V alloy powder than in the other analyzed powders (TiGd1 and Ti-5553). For Ti6Al4V powder, the nitrogen uptake in the surface layer reaches its maximum after 10 h of milling. Moreover, only in this material on the XRD pattern were reflections corresponding to the TiN phase identified, directly confirming the formation of titanium nitride. In addition, after 10 h of milling, the powder particles exhibit a significantly more developed surface morphology, with a sponge-like structure, compared with the other two investigated powders. This phenomenon can be explained by considering the microstructure of the initial powders. In the case of the TiGd1 powder, the microstructure is composed predominantly of the α phase, whereas the Ti-5553 powder exhibits a β-phase structure. However, the Ti6Al4V powder exhibits a martensitic α′ phase that forms during rapid solidification at liquid-cooling rates exceeding 410 °C/s [51]. Such conditions are typically achieved when such a powder is produced by inert gas atomization. The α′ martensitic phase in Ti6Al4V alloy is a supersaturated solid solution of alloying elements formed through a diffusionless β → α′ transformation. This phase is characterized by a high density of defects, including dislocations and twins [52], which facilitate accelerated nitrogen ingress into the material and thereby enhance the nucleation and growth of titanium nitride.

The chemical composition of the powders after grinding was analyzed not only for nitrogen content but also for the presence of elements resulting from contamination with vial material. Even without milling balls, wear of the chamber and lid may introduce Fe/Cr/Ni, which can be important for AM quality, especially for biomaterials-related positioning. Analysis for powders after the longest milling time, 10 h, revealed slight contamination of the titanium alloy powders. However, this problem can be easily eliminated by using ceramic or ceramic-coated cylinders.

## 4. Conclusions

During the experiment, three different materials, such as commercially pure titanium (TiGd1), Ti6Al4V, and Ti-5553 powders, were investigated. It was observed that during self-shearing reactive milling, the nitrogen pressure drop was relatively small in all cases, which suggests that nitriding occurs mainly at the particle’s surface. SEM observations revealed that the titanium nitride layer is very thin and brittle, with a nanometric thickness of approximately 500 nm. The largest nitrogen pressure drop (1.55 bar) after 10 h of milling was exhibited by the Ti6Al4V alloy, and this fact implies that the nitriding effect is more pronounced for this alloy compared to the TiGd1 and Ti-5553 alloy. Elemental analysis confirmed that after 10 h of milling, the Ti6Al4V powder had the highest nitrogen content. From XRD results, only for the Ti6Al4V powder milled for 10 h were the TiN reflections corresponding to the (111) and (220) planes observed, suggesting that the amount of titanium nitride in this case was sufficient to be detected by XRD. Complementary Raman spectroscopy provided further evidence of the existence of titanium nitride TiN on the particle’s surface for all three powders through the identification of characteristic vibrational modes (TA, LA, and TO).

This study revealed that titanium nitride coatings can be deposited on titanium and its alloy powders using a novel process, self-shearing reactive milling (SSRM), under a nitrogen atmosphere. Unlike conventional methods, this approach does not require aggressive environments or elevated temperatures, as the nitriding process occurs at ambient temperature. Moreover, an important aspect of this experiment was the preservation of the spherical shape of the powder particles, as grinding balls were absent during the process. Such powders can be used as a feedstock material for additive manufacturing applications.

## Figures and Tables

**Table 1:** Values of peak positions visible on Raman spectra for pure titanium (TiGd1), Ti6Al4V, and Ti-5553 powders subjected to self-shearing reactive milling under nitrogen pressure.

Sample/Mode	Time of Self-Shearing Reactive Milling					
	Initial Powder	5 min	15 min	30 min	1 h	10 h
**Ti Gd1**						
transverse acoustic (TA)	246	240	224	223	219	203
longitudinal acoustic (LA)	-	321	311	314	310	316
transverse optical (TO)	-	-	577	572	581	564
**Ti6Al4V**						
transverse acoustic (TA)	226	226	223	225	217	176
longitudinal acoustic (LA)	-	314	314	306	306	316
transverse optical (TO)	-	596	601	585	590	580
**Ti-5553**						
transverse acoustic (TA)	-	218	212	216	209	168
longitudinal acoustic (LA)	-	305	302	300	301	299
transverse optical (TO)	-	600	601	602	601	585

## Data Availability

The data presented in this study are openly available in Zenodo at https://doi.org/10.5281/zenodo.18504064.

## Supplementary Materials

The following supporting information can be downloaded at: https://www.mdpi.com/article/10.3390/ma19061115/s1, Figure S1: Particle size distribution histograms of as-received and self-shearing reactive milled Ti, Ti6Al4V, and Ti-5553 powders. The volume-based distributions are shown together with the mean particle size (D_v) and the median particle size (D_v50).; Table S1: Hall flowability results of as-received and self-shearing reactive milled Ti, Ti6Al4V, and Ti-5553 powders.
