# Supplementary material for: Mechanical Nitriding of Titanium and Its Alloys as a Feedstock for the Additive Manufacturing of Functionally Graded Materials
Source: Materials (Basel). 2026 Mar 13;19(6):1115. doi: 10.3390/ma19061115 (PMC13027891; doi:10.3390/ma19061115)
Supplement: Supplementary file 1 [file materials-19-01115-s001.zip › materials-4176918-supplementary.pdf]

Supplementary Materials

Mechanical Nitriding of Titanium and Its Alloys as a Feedstock for the Additive Manufacturing of Functionally Graded Materials

Anna Antolak-Dudka [1], Malwina Liszewska [2], Sławomir Dyjak [3], Iwona Wyrębska [1], Tomasz Czujko [1,*] and Marek Polański [1]

[1] Institute of Materials Science and Engineering, Military University of Technology, Kaliskiego 2, 00-908 Warsaw, Poland; anna.dudka@wat.edu.pl (A.A.-D.); iwona.wyrebska@wat.edu.pl (I.W.); marek.polanski@wat.edu.pl (M.P.)
[2] Institute of Optoelectronics, Military University of Technology, Kaliskiego 2, 00-908 Warsaw, Poland; malwina.liszewska@wat.edu.pl
[3] Institute of Chemistry, Military University of Technology, Kaliskiego 2, 00-908 Warsaw, Poland; slawomir.dyjak@wat.edu.pl
* Correspondence: tomasz.czujko@wat.edu.pl

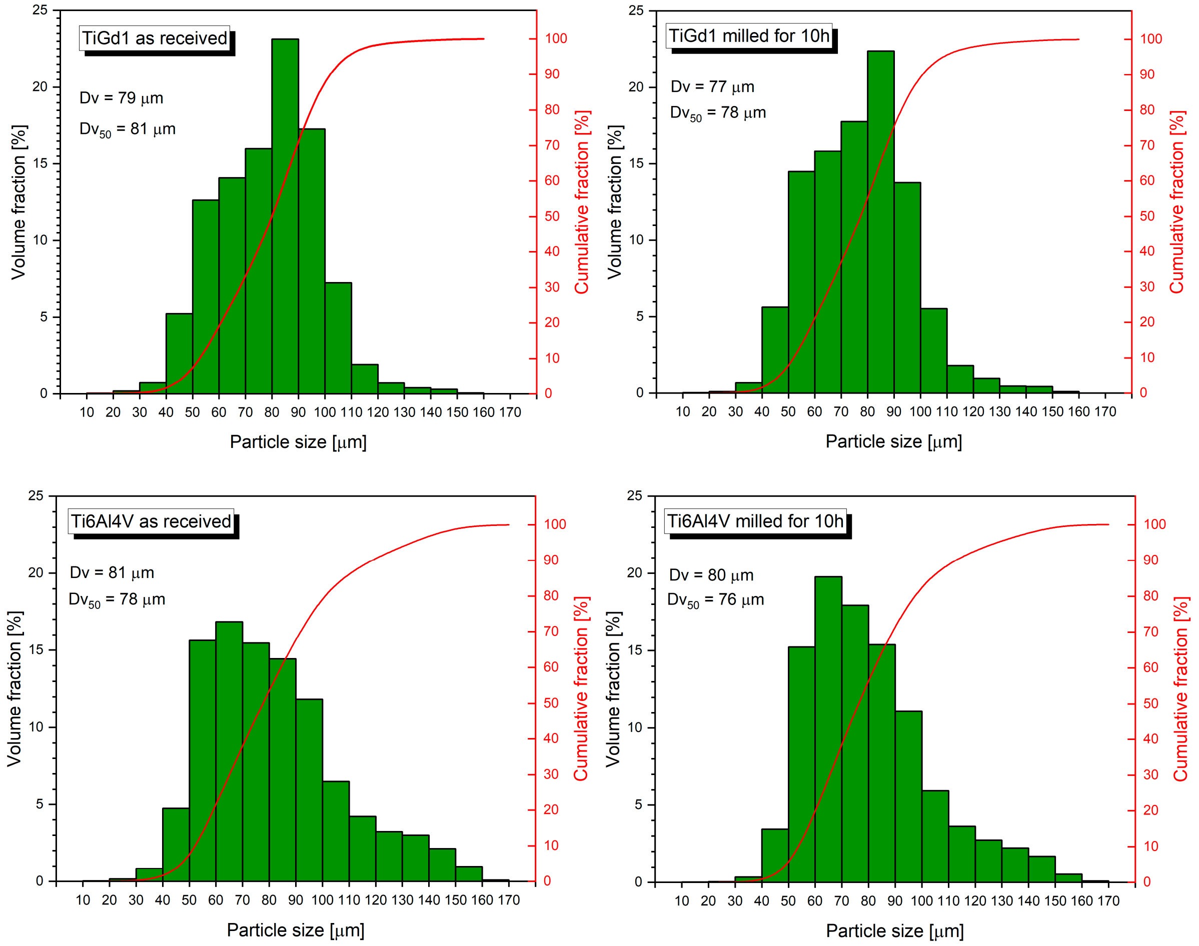

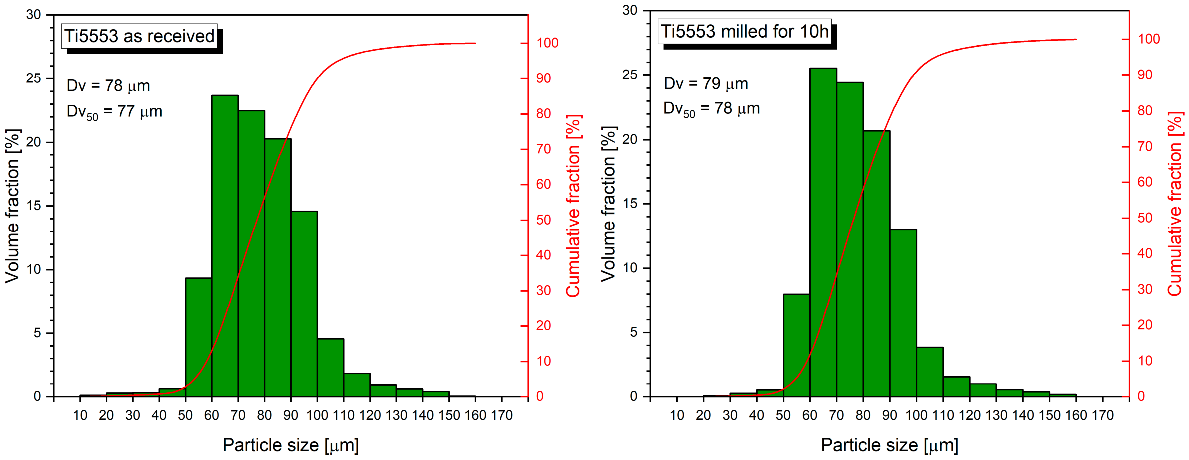

Figure S1. Particle size distribution histograms of as-received and self-shearing reactive milled Ti, Ti6Al4V, and Ti-5553 powders. The volume-based distributions are shown together with the mean particle size (D_v) and the median particle size (D_{v50}).

Table S1. Hall flowability results of as-received and self-shearing reactive milled Ti, Ti6Al4V, and Ti-5553 powders.

	**Flowability [s]**		
	TiGd1	Ti6Al4V	Ti5553
**as received**	26.4 ± 0.1	31.5 ± 0.2	24.7 ± 0.1
**10 h of SSRM**	24.6 ± 0.1	27.3 ± 0.1	24.6 ± 0.1
